# Meta-Analysis and Systematic Review of Phenotypic and Genotypic Antimicrobial Resistance and Virulence Factors in *Vibrio parahaemolyticus* Isolated from Shrimp

**DOI:** 10.3390/antibiotics13040370

**Published:** 2024-04-17

**Authors:** Varangkana Thaotumpitak, Justice Opare Odoi, Saran Anuntawirun, Saharuetai Jeamsripong

**Affiliations:** 1 Department of Microbiology, Faculty of Public Health, Mahidol University, Bangkok 10400, Thailand; varangkana.tha@mahidol.ac.th; 2 Animal Health Division, Animal Research Institute, Council for Scientific and Industrial Research, Accra P.O. Box AH20, Ghana; odoij19890606@gmail.com; 3 Research Unit in Microbial Food Safety and Antimicrobial Resistance, Department of Veterinary Public Health, Faculty of Veterinary Science, Chulalongkorn University, Bangkok 10330, Thailand; pond_saran@hotmail.com

**Keywords:** antimicrobial resistance, resistance determinant, resistance gene, shrimp, *Vibrio parahaemolyticus*, virulence gene

## Abstract

This systematic review and meta-analysis investigates the prevalence of *Vibrio parahaemolyticus*, its virulence factors, antimicrobial resistance (AMR), and its resistance determinants in shrimp. This study was conducted following the Preferred Reporting Items for Systematic Reviews and Meta-Analysis (PRISMA) guidelines, to identify and select relevant peer-reviewed articles published between January 2020 and December 2022. The search strategy involved multiple online databases, including Google Scholar, PubMed, ScienceDirect, and Scopus. The inclusion criteria focused on studies that examined *V. parahaemolyticus* prevalence, virulence factors, and AMR in shrimp from farms to retail outlets. A total of 32 studies were analyzed, revealing a pooled estimate prevalence of *V. parahaemolyticus* in shrimp at 46.0%, with significant heterogeneity observed. Subgroup analysis highlighted varying prevalence rates across continents, emphasizing the need for further investigation. Virulence factor analysis identified thermostable direct hemolysin (*tdh*) and *tdh*-related hemolysin (*trh*) as the most common. Phenotypic AMR analysis indicated notable resistance to glycopeptides, nitrofurans, and beta-lactams. However, the correlation between antimicrobial usage in shrimp farming and observed resistance patterns was inconclusive. Funnel plots suggested potential publication bias, indicating a need for cautious interpretation of findings. This study underscores the urgency of coordinated efforts to address AMR in *V. parahaemolyticus* to safeguard public health and to ensure sustainable aquaculture practices.

## 1. Introduction

Antimicrobial resistance (AMR) has emerged as a critical global health threat, fundamentally reshaping the management of infectious diseases and posing unprecedented challenges to healthcare systems worldwide [1]. The increasing resistance of a diverse array of bacteria responsible for infections in both human and animal populations to previously effective antimicrobial treatments has become a pressing concern [2]. This alarming phenomenon could imperil human health and undermine the sustainability of animal production, and this requires swift and coordinated actions to avert an imminent global health crisis.

*Vibrio parahaemolyticus*, a halophilic bacterium in the family *Vibrionaceae*, thrives within a temperature range from 10 °C to 44 °C, with an optimal growth temperature between 35 °C and 37 °C within marine and estuarine environments [3]. Studies have highlighted the role of this bacterium as a primary causative agent of acute hepatopancreatic necrosis disease in shrimp populations [4]. Further research suggests that *V. parahaemolyticus* can rapidly spread within shrimp tissues, affecting vital organs such as the gills, hepatopancreas, intestine, muscles, and hemolymph, exacerbating the challenges faced in shrimp farming [5]. *V. parahaemolyticus* has the potential to trigger devastating vibriosis outbreaks in shrimp aquaculture, leading to substantial economic losses for farmers worldwide [6]. Historically linked to seafood, such as shrimp, oysters, clams, and cockles, *V. parahaemolyticus* received recognition for its pathogenic potential in humans in 1950 following a major outbreak in Japan in which contaminated sardine with the bacterium led to 20 fatalities and 270 hospitalizations [7]. Subsequently, outbreaks stemming from the consumption of raw or undercooked seafood tainted with *V. parahaemolyticus* have been documented in various countries, including the USA, Thailand, Vietnam, China, Spain, and Chile [8,9,10,11,12,13]. The disease pathogenesis of *V. parahaemolyticus* infections is caused by virulence factors such as thermostable direct hemolysin (TDH) or TDH-related hemolysin (TRH), encoded by *tdh* and *trh* genes, respectively. These two factors are the main factors causing infections in shrimps as well as seafood-borne bacterial gastroenteritis in humans [14]. This emphasizes the significance of comprehending and managing *V. parahaemolyticus* infections to safeguard both the aquaculture industry and the general health of seafood consumers.

Currently, the primary approach to the treatment of *V. parahaemolyticus* infections involves the administration of antimicrobial agents. However, alarming reports have surfaced, highlighting the emergence of AMR strains of *V. parahaemolyticus* and increasing public health concerns [15]. The consequences of infections caused by pathogenic and resistant *V. parahaemolyticus* extend beyond the scope of healthcare, affecting economic and social aspects as well. Outbreaks involving resistant strains can disrupt the seafood industry, erode consumer trust, trigger trade restrictions, and cause economic losses. The interconnectedness between AMR, seafood trade, and economic stability necessitates comprehensive investigations to guide policy interventions and mitigate potential consequences.

The rise of AMR within *V. parahaemolyticus* strains adds further complexity to managing infections, requiring immediate action to mitigate potential impacts on human health and the sustainability of aquaculture systems. The consequences extend to both patient well-being and healthcare systems, leading to prolonged illnesses, treatment failures, and more severe clinical outcomes. Despite its importance, there remains a lack of comprehensive understanding regarding the prevalence of *V. parahaemolyticus*, its virulence factors, and AMR in shrimp, which serve as crucial reservoirs for this pathogen. This study seeks to bridge this gap by conducting a meta-analysis and systematic review of existing literature. The objective of this meta-analysis and comprehensive systematic review was to investigate the prevalence of *V. parahaemolyticus*, along with its virulence factors and AMR phenotype and genotype. This study offers valuable insights into the global occurrence and patterns of AMR in *V. parahaemolyticus* found in shrimp, thereby enhancing the international understanding of foodborne pathogens and AMR. Therefore, the findings from this study can collectively help formulate effective strategies involving various stakeholders to mitigate the impact of AMR in *V. parahaemolyticus*, safeguard public health, and establish resilient socioeconomic systems.

## 2. Results

### 2.1. Summary of Literature Search

**Figure 1:**
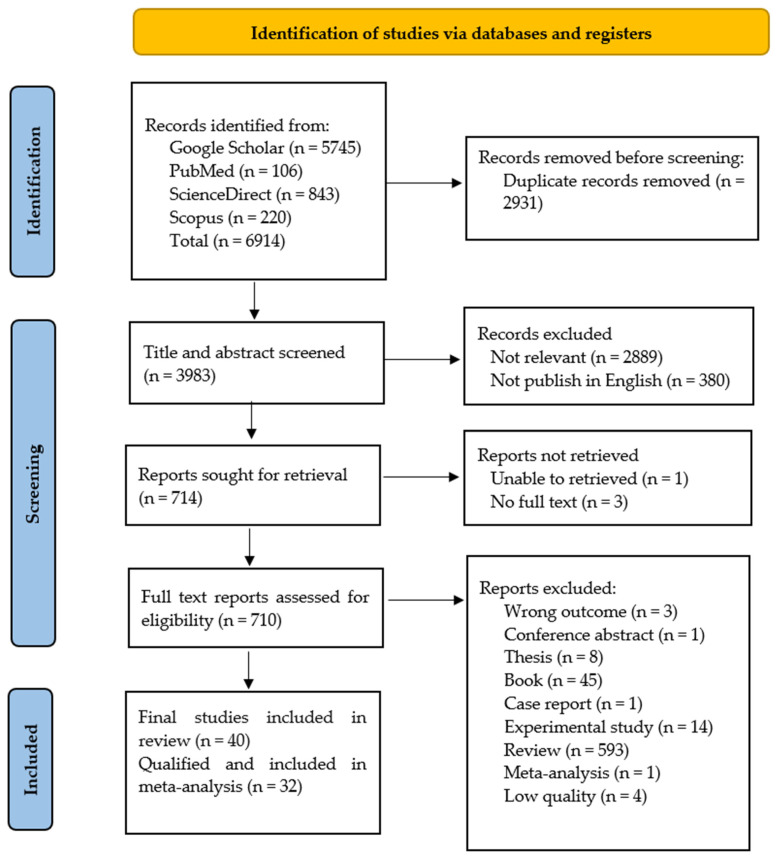
A PRISMA flow chart of the study selection process and the literature search. Different databases were assessed to search for eligible studies of *V. parahaemolyticus* using predefined search strategies.

A total of 6914 articles were initially identified (Figure 1). Following the screening process, 710 articles were selected for further review. Finally, 40 articles were extensively reviewed. However, three of these articles contained few bacterial isolates (*n*~1–3 isolates each), one article presented varying and conflicting counts of isolates, and four articles did not specify the number of positive *V. parahaemolyticus* isolates [16,17,18,19,20,21,22,23].

Therefore, a total of 32 relevant studies from China (*n* = 8), Bangladesh (*n* = 4), Vietnam (*n* = 4), Malaysia (*n* = 4), India (*n* = 4), South Korea (*n* = 2), Cameroon (*n* = 1), Indonesia (*n* = 1), Iran (*n* = 1), Mexico (*n* = 1), Nigeria (*n* = 1), and the United Kingdom (*n* = 1) (Table 1) were selected. Shrimp species such as *Litopenaeus vannamei*, *Penaeus vannamei*, *P. monodon*, *P. merguiensis*, *P. notialis*, *P. kerathurus*, *Parapaeopsis atlantica*, *Metapenaeus ensis*, *M. rosenbergii*, and *Oratosquilla oratoria* were observed in this study.

### 2.2. Bacterial Isolation and Confirmation of V. parahaemolyticus Isolates

Bacterial isolation techniques were based on both national and international standards. The commonly available international standards for detection and confirmation of *V. parahaemolyticus* were the United States Food and Drug Administration Bacteriological Analytical Manual (U.S. FDA BAM), and the International Organization for Standardization (ISO 6222: 1999; ISO 4833-2: 2013; ISO 21872-1: 2017) [54,55,56]. The national standards included the Nation Standard Food Microbiological Examination *V. parahaemolyticus* (GB 4789.7-2013) and the Indonesia National Standard (SNI) 01-2332.5-2006, and available published papers, including Oanh et al. (2018), Blanco-Abad et al. (2009), Cowan and Steel’s Manual, Barrow and Feltham (1993), and Bergey (2005) in some studies [57,58,59,60,61,62]. Thiosulfate–citrate–bile salts–sucrose agar (TCBS) and Chromogenic agar were the most common media used for bacterial isolation.

For *V. parahaemolyticus* confirmation, colony morphology, Gram staining, and biochemical tests (namely, acetyl methyl carbinol production test, arginine dihydrolase test, catalase test, citrate test, dextrose utilization test, gelatinase test, growth at 0, 3, 6, 8, and 10% NaCl concentrations, growth at 4, 20, 30, 35, and 40 °C, halophilism test, hemolysis test, hydrogen sulfide production test, luminescent bacterial test, lysine decarboxylase test, methyl red (MR) test, Voges–Proskauer (VP) test, motility test, oxidase test, O-nitrophenyl beta-D-galactosidase (ONPG) test, ornithine decarboxylase test, indole test, oxidation fermentation (O-F) test, salt tolerance test, sulfur reduction (cysteine desulfurase) test, utilization of alpha ketoglutarate, urease test, triple sugar iron agar (TSI), as well as the commercial API 20E biochemical test kits (bioMérieux, Marcy-l’Etoile, France) were applied. In addition to PCR, MALDI-TOF and VITEK2 were employed by the studies for bacterial identification. Furthermore, 16S rRNA sequencing along with species-specific genes (*tlh, tl*, and *tox*R) detection were also utilized.

### 2.3. Prevalence of V. parahaemolyticus and Virulence Factors

The prevalence of *V. parahaemolyticus* in different anatomical parts of shrimp, such as hepatopancreas, hemolymph, gill, intestine, midgut, stomach, tissue or flesh, whole shrimp, and swab samples, were considered in the analysis. In addition to shrimp, some studies included other aquatic animals such as marine fish, oysters, crabs, shellfish, clams, mussels, tilapia, rui, and squid for examination. A subset of studies also included environmental samples, such as sediment, pond water, and reservoir water.

**Figure 2:**
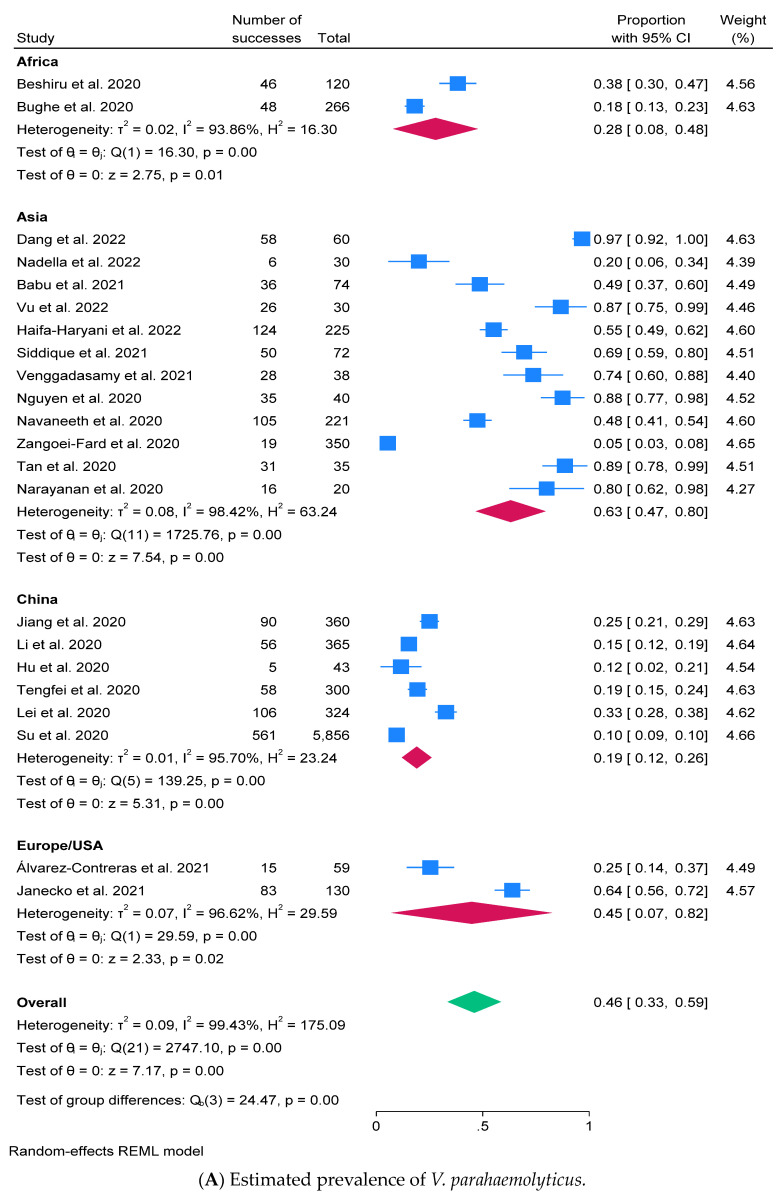

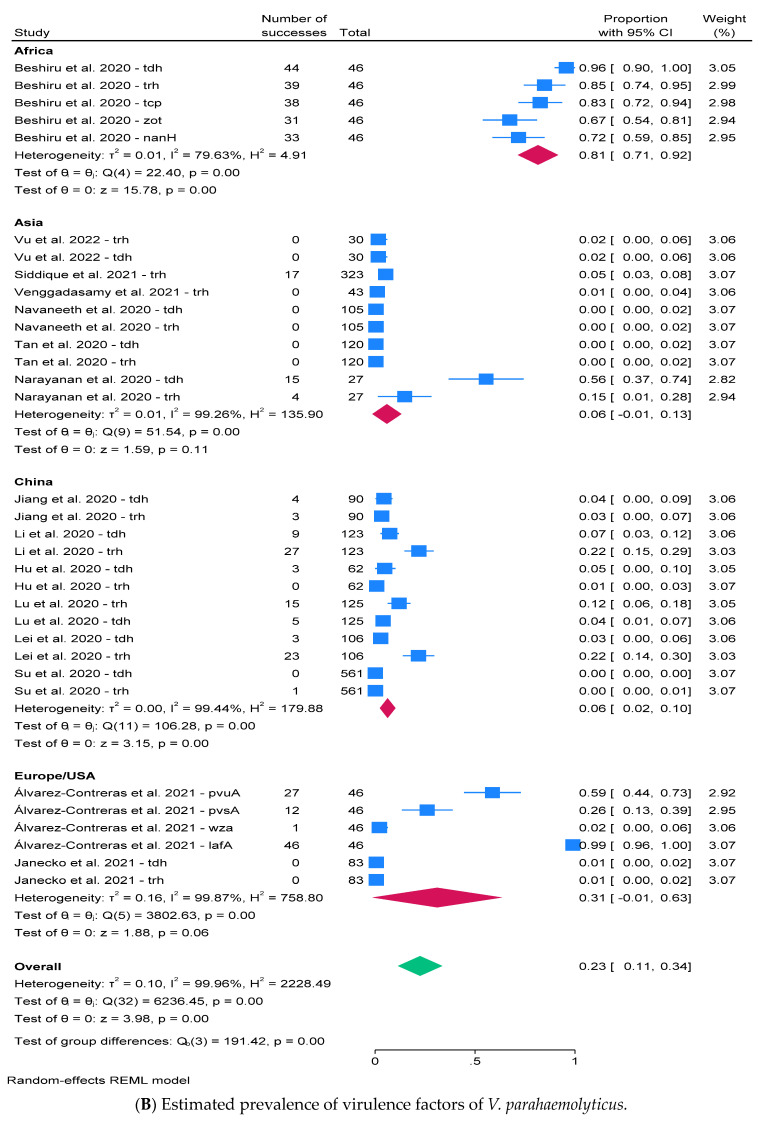

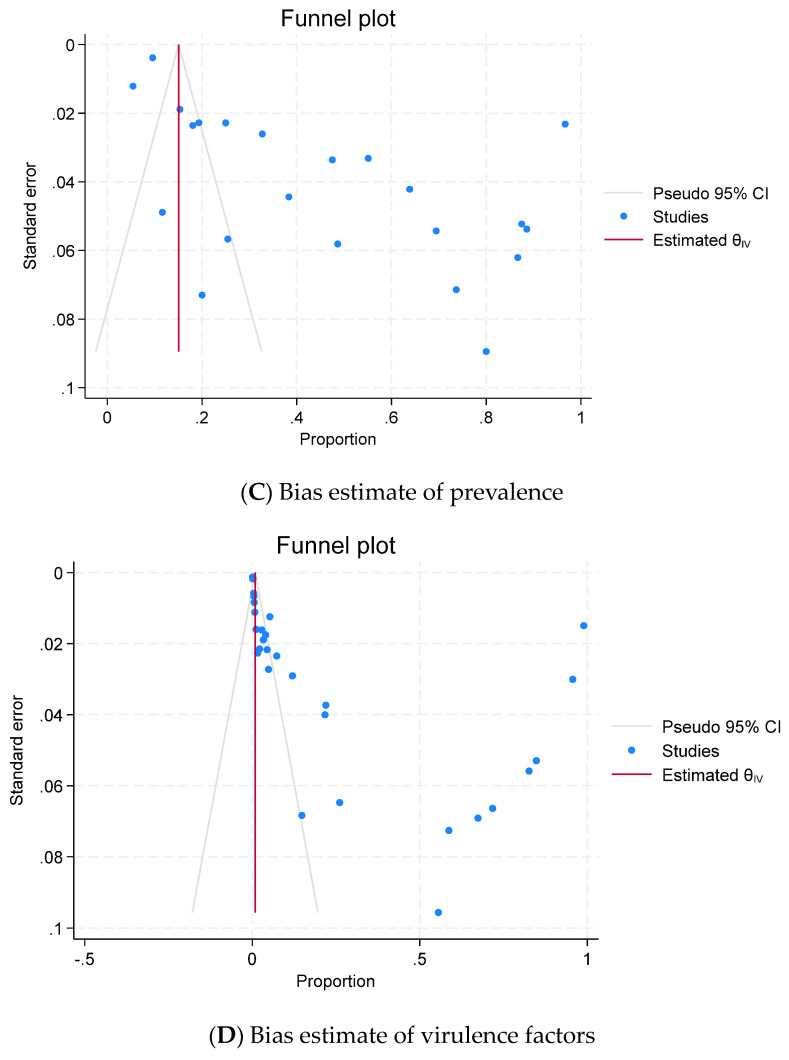
The meta-analysis of prevalence of *V. parahaemolyticus* and virulence factors in shrimp using random-effects model with a 95% C.I. A forest plot of (**A**) prevalence of *V. parahaemolyticus* [19,25,26,27,28,29,31,33,34,35,36,37,38,39,40,41,42,43,44,46,47,48,49,51,53] and (**B**) prevalence of virulence factors [4,24,28,33,34,35,36,38,39,40,42,44,45,47,49,51,52,53] representing the results in meta-analysis with size squares proportional to the weight assigned to the study; a funnel plot of the prevalence estimates of *V. parahaemolyticus* (**C**) and virulence factors (**D**) to assess potential bias. A red vertical line represents the overall prevalence from meta-analysis, and the diagonal lines provide 95% C.I. The prevalence with ranges of individual virulence factors is represented by the light blue square with line, while the average prevalence is presented by the red diamond. The overall heterogeneity of virulence factors is indicated by the green diamond.

This comprehensive approach allowed for a robust examination of *V. parahaemolyticus* prevalence, considering both the random-effects model and the visualizations of study weights and bias assessment. The prevalence of *V. parahaemolyticus* was determined among 22 studies (22/32 = 68.8%) from 9018 samples/isolates, and the pooled estimate of *V. parahaemolyticus* prevalence in shrimp was 46.0% with a 95% C.I. (33.4–58.6%) and Q-statistic 2747.10 (Figure 2A). The overall heterogeneity was identified with tau^2^ = 0.088, I^2^ = 99.43%, H^2^ = 175.09, and *p*-value < 0.0001.

Although our study primarily provided descriptive presentations of the prevalence, virulence factors, and AMR of *V. parahaemolyticus* in shrimp populations, we recognize the potential advantages of conducting further analyses to extract additional insights from the dataset. As such, we have performed subgroup analyses to explore the relationship between variables more comprehensively and to identify potential determinants of *V. parahaemolyticus* prevalence and AMR in shrimp across different continents. Subgroup analysis was assessed based on continents—except China due to availability of several publications. The prevalence of *V. parahaemolyticus* was high in Asia (63.1% with C.I. = 46.7–79.5%), while China exhibited the lowest prevalence at 19.0% (C.I. = 12.0–26.0%). A funnel plot was employed for bias assessment, contributing to a comprehensive evaluation of the prevalence data (Figure 2C).

The analysis of virulence genes in *V. parahaemolyticus* within shrimp was conducted in 15 out of 32 articles (46.9%) from 3644 bacterial isolates. Virulence factors were limited to a specified set that included *tdh*, *trh*, *pvuA*, *pvsA*, *wza*, *lafA*, *tcp*, *zot*, *nanH*, *pirA*, and *pirB*. Among these, *tdh* and *trh* were identified as the most frequently detected virulence genes. The estimated prevalence of virulence factors was determined as 22.5% with a 95% C.I. of 11.4–33.6% (Figure 2B). The heterogeneity was observed using a Q value of 6236.45 with tau^2^ = 0.104, I^2^ = 99.96%, H^2^ = 2228.49, and *p*-value < 0.0001 (Figure 2B), and the bias assessment was indicated in a funnel plot (shown here in Figure 2D).

### 2.4. Antimicrobial Susceptibility Testing (AST)

Among 32 studies used (*n* = 29/32, 90.6%), the phenotypic antimicrobial susceptibility of *V. parahaemolyticus* was mostly tested by disk diffusion test (*n* = 23), broth microdilution method (*n* = 5), and both disk diffusion and broth microdilution (*n* = 1). The methods used for AST were followed by the Clinical and Laboratory Standards Institute (CLSI) (EP17, M45, and M100), European Committee on Antimicrobial Susceptibility Testing (EUCAST), and National Committee for Clinical Laboratory Standards (NCCLS). The interpretive criteria were as indicated according to the CLSI breakpoints and epidemiological cut-off values (ECOFFs) set by EUCAST.

Additionally, *Escherichia coli* ATCC 25922 (*n* = 12), *V. parahaemolyticus* ATCC 17802 (*n* = 2), both *V. parahaemolyticus* ATCC 17802 and 33847 (*n* = 2), both *E. coli* ATCC 25922 and *V. parahaemolyticus* ATCC 17802 (*n* = 1), both *E. coli* ATCC 25922 and *Staphylococcus aureus* ATCC 29213 (*n* = 1), *V. parahaemolyticus* DSM 11058 (*n* = 1), and *V. parahaemolyticus* (MTCC451, IMTCC, Chandigarh, India) (*n* = 1) were used as bacterial control strains, while 11 published studies did not mention any bacterial control strains.

### 2.5. Prevalence of AMR and Resistant Determinants

The occurrence of AMR in *V. parahaemolyticus* was investigated across 2164 isolates from 25 studies focusing on 16 antimicrobial classes. These antimicrobial classes comprised aminocyclitol, aminoglycosides, beta-lactams, carbapenems, cephalosporins, folate pathway inhibitors/sulfonamides, glycopeptides, lincosamides, macrolides, nitrofurans, oxazolidines, phenicols, polypeptides, quinolones, rifampicins, and tetracyclines (Table 2). A limited number of studies examined AMR in aminocyclitol (*n* = 1), glycopeptides (*n* = 3), lincosamides (*n* = 1), nitrofurans (*n* = 3), oxazolidines (*n* = 1), and rifamycins (*n* = 1).

The pooled estimate prevalence of phenotypic AMR in *V. parahaemolyticus* was categorized based on antimicrobial classes (Table 2). The predominant resistance phenotype examined was resistance to glycopeptides (i.e., vancomycin, novobiocin) (60.7% with 95% C.I. 19.6–101%, *p* < 0.0001), followed by nitrofurans (e.g., nitrofurantoin) (58.8% with 95% C.I. 17.8–99.8%, *p* < 0.0001), beta-lactams (e.g., amoxicillin, ampicillin, penicillin, etc.) (56.0% with 95% C.I. 44.6–67.5%, *p* < 0.0001), and polypeptides (e.g., bacitracin, colistin, polymyxin B) (31.2%, 95% C.I. 5.7–68.2%, *p* < 0.0001). However, exploration of resistance to carbapenems in China, folate pathway inhibitors/sulfonamides in Africa, Europe, and USA, and nitrofurans, aminocyclitol, and rifampicins at a global level was limited. The identification of bias was also conducted by examining funnel plots stratified by antimicrobial classes (see Figure S1).

Regarding analysis of AMR genes, one study from India conducted AMR genotypes of phenicols and tetracyclines [26]. Overall prevalence of phenicol resistance genes, including *cat*I, *cat*II, and *flor*R, was estimated at 11.3% with a 95% C.I. of 3.3–19.3%, *p* = 0.865, while the higher prevalence was observed in tetracycline resistance genes (*tet*A, *tet*B, *tet*K, *tet*L, *tet*M, and *tet*S) at 18.1% with a 95% C.I. of 5.2–31.0%, *p* = 0.0191. In the same study, the estimated prevalence of mobile genetic elements was also observed to be 14.6% with 95% C.I. 3.5–25.7%, and the genes *int*1, *int*2, *Tn*1720, *Tn*1545, and *Tn*917 were examined. A bias assessment was conducted (Figure S2).

### 2.6. Publication Bias (Reporting Bias)

In this meta-analysis, publication bias was graphically assessed using visual funnel plots by the regression-based test of Egger at *p* < 0.05. On visual inspection, the funnel plot showed slight asymmetrical distribution in meta-analysis for *V. parahaemolyticus*, virulence factors, AMR, and their determinants (Figure 2C,D, Figures S1 and S2).

## 3. Discussion

This study comprehensively investigated the overall prevalence of *V. parahaemolyticus*, virulence factors, and AMR isolates in shrimp retrieved from 32 published articles. Given the considerable diversity in study designs and population characteristics across the included studies, a random-effects model was employed for meta-analysis. This study involved the inclusion of equal proportions of both retail and agricultural farms. The examination of subgroup meta-analysis was conducted with a focus on continents, revealing a heightened prevalence of *V. parahaemolyticus* in Asia at 57.0%, with China exhibiting the lowest prevalence at 19.0%. Nevertheless, there was a scarcity of studies from Europe and the USA. Shrimp presented a higher risk, with a prevalence rate of 3.2%, when contrasted with other decapod crustaceans such as lobster and crab, which had prevalence rates of 3.0% and 3.1%, respectively [63]. Consequently, it is imperative to conduct additional research to evaluate the prevalence of *V. parahaemolyticus* and its virulence factors, enhancing the representativeness of findings across these continents. The assessment of virulence factors in shrimp revealed that the predominant virulence factors detected were *tdh* and *trh*. Additionally, five studies performed serotyping for *V. parahaemolyticus* isolates, and the distribution of serotypes O3:K6, O5:KUT, O11:KUT, O3:KUT, O1:KUT, O3:K20, O1-O8, O10-O12, K25, K31, K64, and K68 was observed [33,42,44,48,49].

The primary technique used for AST involved a disk diffusion test, followed by a broth microdilution test. Notably, in South Korea, an automatic system such as Sensititre™ was used to perform broth microdilution [30,31]. The criteria for choosing antimicrobials for AST were not clearly outlined. However, it is recommended that antimicrobials should be selected based on AMR monitoring and surveillance guidelines by following CLSI standards for aquatic animals, humans, and animals and the OIE list of antimicrobial agents of veterinary importance [64,65,66]. While *E. coli* ATCC 25922 serves as the primary control strain for AST, about one-third of the studies did not explicitly mention the use of reference strains. This lack of clarity raises concerns about the reliability of the results in those studies, emphasizing the importance of proper reference controls in ensuring the accuracy of antimicrobial susceptibility assessments. In all the studies reviewed, the primary molecular method used for identifying resistance genes was predominantly PCR. However, the use of Whole-Genome Sequencing (WGS) was limited and not extensively utilized for this purpose.

This study observed that *V. parahaemolyticus* predominantly exhibited phenotypic resistance to glycopeptides, nitrofurans, and beta-lactams, a pattern similar to findings in a study on AMR *Vibrio* in marine bivalves [67]. Notably, these antimicrobials are not commonly used in shrimp farming practices. Contrasting results were found in studies on shrimp aquaculture in China and Vietnam, where oxytetracycline emerged as the primary antimicrobial and frequently used antimicrobial classes included tetracyclines, sulfonamides, and quinolones [68,69]. The inconsistency in these observations tentatively suggests that phenotypic AMR may not necessarily arise because of the selective pressure of antimicrobial applications on farms. Moreover, *V. parahaemolyticus* isolates with high resistance were not detectable in shrimp feed, implying a potential contribution from external sources of antimicrobials entering the farms. In human medicine, glycopeptides, nitrofurans, and beta-lactams are widely prescribed, and their resistance is observed in hospital wastewater worldwide [70]. To address the potential consumer risk associated with shrimp consumption, further studies are needed to investigate the sources of AMR contamination in shrimp farms, particularly focusing on environmental water.

Funnel plots were performed to evaluate publication bias and heterogeneity. Larger sample studies are clustered near the mean effect size at the top of the graph, contrasting with smaller-sample studies positioned at the bottom. The variability in effect size estimates within these smaller studies was influenced by sampling differences, evident in the graph’s distribution. Asymmetry in funnel plots indicated potential publication bias for the prevalence estimate of *V. parahaemolyticus*, virulence factors, and AMR. Notably, the funnel plot for *V. parahaemolyticus* prevalence suggests more heterogeneity with varied sample sizes than that from the occurrence of virulence factors. Regarding the funnel plot for *V. parahaemolyticus* prevalence, smaller studies with significant results were more likely to be published than those without significant virulence factors exhibited.

The previous studies referred to the CLSI standard for AST. Most of these studies adopted CLSI criteria originally developed for human and animal sources. Notably, only one CLSI document suggested nine antimicrobial classes for susceptibility tests specific to *V. cholerae* and other *Vibrio* spp. These classes included aminoglycosides, beta-lactams, carbapenems, cephalosporins, folate pathway inhibitors, macrolides, phenicols, quinolones, and tetracyclines with limited reference ranges [71]. Recent CLSI guidelines for bacteria isolated from aquatic animals do not provide any reference criteria for *Vibrio* spp. [65]. One study specifically examined the resistance of *V. parahaemolyticus* to medemycin, a newly developed macrolide antibiotic. This was because of the increased use of this drug due to its higher potency and low gastrointestinal irritation compared with other macrolides [72]. Results in our study suggested that harmonized protocols for antimicrobial resistance testing in aquaculture should be promptly initiated to achieve a valid global AMR assessment and prioritize urgent needs.

The limitations of this study were classified into two primary aspects: the review process and the evidence included in the review. The comprehensiveness of this study may be influenced by the chosen search strategies and time constraints. The potential introduction of bias could occur if other articles were not included in the study. Regarding the evidence incorporated in this study, the reliability of the included articles may exhibit variation concerning study design, sampling methods, and the approach to bacterial identification and selection criteria for performing AST. These variations could influence the overall robustness of the review and present challenges in the analysis of the data. Therefore, it is important to expand the pool of studies and continuously monitor the trends in *V. parahaemolyticus* and its resistant isolates. Further investigation in this regard is necessary to enhance our understanding and inform ongoing efforts to address potential public health implications.

In future research, it is essential to monitor and survey the trends in resistant *V. parahaemolyticus* isolates. This is crucial for evaluating the effectiveness of national and international frameworks or policies addressing AMR within the aquaculture sector. Such trend data derived from epidemiological studies will also contribute to a better understanding of the situation.

## 4. Materials and Methods

### 4.1. Study Selection

This systematic review was conducted following the Preferred Reporting Items for Systematic Reviews and Meta-Analysis (PRISMA) guidelines (Table S1) [67]. Different peer-reviewed journal articles published between 1 January 2020 and 31 December 2022 were selected based on inclusion and exclusion criteria. The prevalence of *V. parahaemolyticus* was calculated as the proportion of positive samples to the total number of samples, and the occurrence of virulence factors and AMR was expressed as the proportion of positive isolates to the total number of bacterial isolates. The primary focus of this study was on determining the prevalence of *V. parahaemolyticus* in shrimp from farms through retail outlets. Moreover, because of the significant losses in shrimp production caused by *V. parahaemolyticus*, only analyses involving this organism were taken into account, and data on any other aquatic animals were disregarded. This investigation specifically characterized the phenotypic and genotypic profiles of AMR, virulence factors, and resistance determinants in *V. parahaemolyticus* isolates obtained from these shrimp samples. To ensure the inclusion of publications, we implemented control measures involving cross-referencing between Web of Science (https://mjl.clarivate.com/search-results, accessed on 13 April 2024) and other databases. Furthermore, the words used in identification algorithms were clear and unique, increasing the likelihood of retrieving overlapping publications.

### 4.2. Eligibility Criteria

The articles that were considered eligible were peer-reviewed research papers published in reputable scientific journals listed in Google Scholar (https://scholar.google.com/, accessed on 13 April 2024), PubMed (https://pubmed.ncbi.nlm.nih.gov/, accessed on 13 April 2024), ScienceDirect (https://www.sciencedirect.com/, accessed on 13 April 2024), and Scopus (https://www.scopus.com/, accessed on 13 April 2024). All articles focused on pathogenicity and AMR characterization in *V. parahaemolyticus* as significant aspects of the study. The studies concerned strains of *V. parahaemolyticus* isolated from shrimp with exposure to AMR patterns, particularly phenotypic (e.g., disk diffusion and dilution methods) and genotypic (e.g., detection of resistance genes and determinants) assessments. Finally, the articles that were considered eligible were accessible for detailed analysis and reporting standards, including providing proper bacterial isolates; clear descriptions of the methods for bacterial isolation and confirmation, the method of antimicrobial susceptibility testing (AST), and the detection of virulence factors; and the results, discussions, and conclusions could be followed. These criteria were consistently applied during the article selection process.

### 4.3. Inclusion Criteria

This systematic review specifically targeted shrimp as the animal species of interest, with a primary focus on examining the presence of *V. parahaemolyticus* in shrimp. Included studies were those that examined the prevalence of virulence genes, AMR, or/and the associated resistance determinants in *V. parahaemolyticus* isolates derived from shrimp. Various methods were employed for the detection and confirmation of *V. parahaemolyticus*, including bacterial culture, biochemical tests, analytical profile index (API20E), and molecular techniques such as polymerase chain reaction (PCR) and Whole-Genome Sequencing. The AST method for detecting AMR phenotypes was also considered. Additionally, molecular techniques for identifying virulence genes, resistance genes, and their determinants were incorporated. The studies were limited to studies published in English.

### 4.4. Exclusion Criteria

Other aquatic animals, such as bivalves, fish, mollusks, and aquatic plants, were excluded from this study. Studies that did not report sample size, number of isolates, the method for bacterial isolation and confirmation, and AST were excluded from this study. Antimicrobials, including antifungals, antiseptics, and disinfectants, were also excluded from this study. Questionnaires, survey studies, and epidemiological studies without aquatic animals were also excluded. Studies that were not primary or original research but were based on review articles, abstracts, proceedings, short communications, correspondence, editorials, opinion articles, research theses, and book chapters were excluded. In addition, studies that were not published in the English language and were non-peer reviewed were not included.

### 4.5. Search Strategy

The search strategy was to explore the prevalence of bacteria, virulence factors, AMR, and resistant determinants among *V. parahaemolyticus* isolated from shrimp samples. Either the presence or absence of virulence factors, AMR, and resistant determinants of *V. parahaemolyticus* isolates was considered possible in the eligible criteria. The online databases searched were Google Scholar, PubMed, ScienceDirect, and Scopus. Searches on commercial websites, including Google and Yahoo, were performed using Boolean operators such as AND, OR, and NOT in conjunction to combine keywords in database searches (Table S2) [73].

The identification algorithm for the relevant literature was listed using the following keywords: *V. parahaemolyticus*, antimicrobial resistance, AMR, antibiotic resistance, AST, resistance profile, virulence factors (TDH, TRH, and other pathogenicity), and resistance determinants (integrons, integrative and conjugative elements (ICEs), and plasmid), specifically in shrimp, from farm to retail. Several keyword combinations were also used for each topic area to ensure as many articles as possible were captured within the specified timeframe and subject area.

This systematic review followed the PRISMA guidelines and a completed PRISMA 2020 checklist is provided (Table S1). Complete publications were reviewed by three veterinarians and one microbiologist. Any discrepancies or uncertainties were resolved through discussion and consensus among the reviewers. Additionally, we ensured the verification and credibility of the selected articles by cross-referencing them with reputable scientific journals listed in the databases. Subsequently, all articles that met the inclusion criteria were consolidated, and duplication detection was performed. Publications that met the inclusion criteria were classified by bacterial isolation and confirmation of *V. parahaemolyticus* from shrimp, number of total and positive samples and isolates, country, method of AST, and molecular detection of resistance genes and their determinants. All data with full details of the search strategy and results were independently extracted from materials and methods, results, discussions, figures, and tables by two individuals (V.T. and S.A.) and double-checked by the third investigator (S.J.), and the qualified articles were compiled in Microsoft Excel for further data analysis.

### 4.6. Data Collection and Assessment of Data Quality

Inclusion and exclusion criteria were used to detect the possible publications relevant to this study. The primary outcome measures were based on the prevalence of *V. parahaemolyticus* and contained virulence factors, phenotypic and genotypic AMR, and other genetic determinants. All eligible published articles underwent extraction, encompassing essential details such as the article title, author names, publication year, country, duration of data collection, shrimp species, shrimp status, total samples/bacterial isolates, number of positive samples/isolates, virulence genes, resistance phenotypes and genotypes, as well as their determinants. Additionally, other variables included were the detection and confirmation of *V. parahaemolyticus*, method for AST, bacterial control strains, and molecular techniques used for detection of resistance genes and their genetic determinants.

### 4.7. Statistical Analyses

The extracted data were stored in a Microsoft Excel spreadsheet and imported into STATA program version 18.0 (StataCorp, College Station, TX, USA). An observational study for one-sample binary outcome summaries of the prevalence of *V. parahaemolyticus*, virulence factors, AMR, and their determinants isolated from shrimp was examined. The prevalence was calculated based on the proportion of positive samples/isolates to number of total samples/isolates. The data are expressed as a pooled estimate of the prevalence of *V. parahaemolyticus*, virulence factors, resistance to each antimicrobial class, and determinants. Random-effects meta-analyses were conducted, and the summary weighted average proportion (effect size) was calculated based on the individual effect sizes and 95% confidence intervals (C.I.). Heterogeneity between studies of prevalence estimates among the studies was investigated using Q-statistics with I^2^ Index [74,75]. A possible source of heterogeneity among observed studies was assessed through subgroup analysis based on the continent of the source of samples.

The visual assessment of publication or dissemination bias was performed using a funnel plot for asymmetry with a pooled estimate. Egger’s tests were employed for detecting publication bias. Studies were included in the meta-analyses if they met the criteria, including at least 20 isolates for all analyzed studies and at least three studies for each meta-analysis. All analyses were two-tailed tests, with statistical significance set at a *p*-value < 0.05. The meta-analyses utilized a random-effects model in STATA software.

## 5. Conclusions

In conclusion, this comprehensive systematic review and meta-analysis has presented the prevalence of *V. parahaemolyticus* in shrimp, along with its associated virulence factors and AMR profiles. The study revealed a significant prevalence of *V. parahaemolyticus* in shrimp samples globally, with Asia exhibiting particularly high rates. Notably, virulence genes, such as *tdh* and *trh*, were frequently identified, indicating the potential pathogenicity of *V. parahaemolyticus*. Our study highlighted the levels of phenotypic AMR, revealing significant resistance against glycopeptides, nitrofurans, and beta-lactams. Furthermore, the investigation identified gaps in AMR surveillance and control measures, emphasizing the need for harmonized protocols and enhanced monitoring efforts. This finding showed the importance of ongoing research and collaborative interventions to address the growing threat of AMR in *V. parahaemolyticus*, to ensure public health safety, and to promote sustainable aquaculture practices.

## Figures and Tables

**Table 1:** Descriptive summary of prevalence of *V. parahaemolyticus* and virulence genes among relevant studies (*n* = 32).

ID	Author and Published Year	Country	Study Period	Bacteria			Virulence			
				TS	PS	P (%)	Gene	TS	PS	P (%)
1	Mulya et al. (2022) [24]	Indonesia	NA	NA	12	NA	*tdh* *trh*	12 12	12 0	100.0 0
2	Dang et al. (2022) [25]	Vietnam	NA	60	58	96.7	NA	NA	NA	NA
3	Nadella et al. (2022) [26]	India	January 2017–December 2018	30	6	20.0	NA	NA	NA	NA
4	Babu et al. (2021) [27]	India	February 2014–July 2015	74	36	48.6 *	NA	NA	NA	NA
5	Vu et al. (2022) [28]	Vietnam	May 2020–October 2020	30	26	86.7	*tdh* *trh*	30 30	0 0	0 0
6	Haifa-Haryani et al. (2022) [29]	Malaysia	March 2019–March 2021	225	124	55.1	NA	NA	NA	NA
7	Kim et al. (2021) [30]	South Korea	Autumn 2016	NA	48	NA	NA	NA	NA	NA
8	Mok et al. (2021) [31]	South Korea	April 2018–November 2018	11	6	54.5 **	NA	NA	NA	NA
9	Jin et al. (2021) [32]	China	2015–2016	180	NA	NA	NA	NA	NA	NA
10	Siddique et al. (2021) [33]	Bangladesh	May 2017–April 2018	72	50	69.4	*trh*	323	17	5.3
11	Álvarez-Contreras et al. (2021) [34]	Mexico	August 2017–February 2018	59	15	25.4	*pvuA* *pvsA* *wza* *lafA*	46 46 46 46	27 12 1 46	58.7 26.1 2.2 100.0
12	Janecko et al. (2021) [35]	United Kingdom	May 2018–April 2019	130	83	63.8 *	*tdh* *trh*	83 83	0 0	0 0
13	Venggadasamy et al. (2021) [36]	Malaysia	NA	38	28	73.7	*trh*	43	0	0
14	Yasin et al. (2021) [19]	Bangladesh	NA	13	1	7.7	NA	NA	NA	NA
15	Nguyen et al. (2020) [37]	Vietnam	March 2018–June 2018	40	35	87.5	NA	NA	NA	NA
16	Navaneeth et al. (2020) [38]	India	April 2013–March 2017	221	105	47.5	*tdh* *trh*	105 105	0 0	0 0
17	Jiang et al., 2020 [39]	China	2017–2019	360	90	25.0	*tdh* *trh*	90 90	4 3	4.4 3.3
18	Beshiru et al., 2020 [40]	Nigeria	November 2016–December 2017	120	46	38.3	*tdh* *trh* *tcp* *zot* *nanH*	46 46 46 46 46	44 39 38 31 33	95.7 84.8 82.6 67.4 71.7
19	Rahman et al., 2020 [41]	Bangladesh	NA	16	4	25.0	NA	NA	NA	NA
20	Li et al., 2020 [42]	China	September 2015–March 2016	365	56	15.3	*tdh* *trh*	123 123	9 27	7.3 22.0
21	Zangoei-Fard et al., 2020 [43]	Iran	October 2017–October 2018	350	19	5.4	NA	NA	NA	NA
22	Hu et al., 2020 [44]	China	June 2018–October 2018	43	5	11.6	*tdh* *trh*	62 62	3 0	4.8 0
23	Lu et al., 2020 [45]	China	April 2014–December 2015	NA	NA	NA	*tdh* *trh*	125 125	15 5	12.0 4.0
24	Bughe et al., 2020 [46]	Cameroon	May 2014–April 2015	266	48	18.1	NA	NA	NA	NA
25	Tan et al., 2020 [47]	Malaysia	Jane 2018–June 2018	35	31	88.6	*tdh* *trh*	120 120	0 0	0 0
26	Hong To et al., 2020 [4]	Vietnam	2015–2017	NA	NA	NA	*pirA* *pirB*	12 12	10 12	83.3 100
27	Tengfei et al., 2020 [48]	China	January 2017–December 2019	300	58	19.3	NA	NA	NA	NA
28	Lei et al., 2020 [49]	China	June 2014–June 2015	324	106	32.7	*tdh* *trh*	106 106	3 23	2.8 21.7
29	Amin et al., 2020 [50]	Bangladesh	NA	NA	NA	NA	NA	NA	NA	NA
30	Narayanan et al., 2020 [51]	India	NA	20	16	80	*tdh* *trh*	27 27	15 4	55.6 14.8
31	Amatul-Samahah et al., 2020 [52]	Malaysia	May 2017	NA	NA	NA	*tdh* *trh* *pirA* *pirB*	2 2 2 2	0 0 2 2	0 0 100 100
32	Su et al., 2020 [53]	China	July 2017–August 2017	5856	561	9.6	*tdh* *trh*	561 561	0 1	0 0.2

TS: total samples; PS: positive samples; P: prevalence; NA: data not available; *: data based on number of isolates; **: data based on number of farms.

**Table 2:** Summary of pooled prevalence of AMR *V. parahaemolyticus* stratified by antimicrobial classes.

Antimicrobial Class	Antimicrobials	Total Tested Isolates	Pooled Prevalence (%)	95% C.I.	tau^2^	I^2^ (%)	H^2^ (%)	*p*-Value
Aminocyclitol *	Spectinomycin	561	NA					
Aminoglycosides	Amikacin; gentamicin; kanamycin; piperacillin; streptomycin; netilmicin; neomycin; tobramycin	5049	21.7	14.0–29.3	0.070	99.57	230.44	<0.0001
	Africa	264	1.7	0.2–3.2	0.0	0.11	1.00	0.017
	Asia	1537	15.1	5.5–24.7	0.052	99.51	204.6	<0.0001
	China	3110	33.7	18.3–49.1	0.098	99.63	272.35	<0.0001
	Europe/USA	138	38.0	20.3–55.7	0.020	80.67	5.17	0.004
Beta-lactams	Amoxicillin; ampicillin; ampicillin-sulbactam; carbenicillin; penicillin; amoxicillin/clavulanic acid; carboxybenzicillin; oxacillin; piperacillin; piperacillin/tazobactam; ticarcillin	3842	56.0	44.6–67.5	0.145	99.77	442.14	<0.0001
	Africa	270	50.6	21.5–79.7	0.129	97.87	46.91	<0.0001
	Asia	1861	52.2	34.4–70.1	0.182	99.87	755.17	<0.0001
	China	1619	63.7	46.1–81.3	0.103	99.53	213.54	<0.0001
	Europe/USA	92	63.9	−5.4–133.2	0.247	99.07	107.85	<0.0001
Carbapenems	Imipenem; meropenem; tebipenem	824	0.7	0.01–1.3	0	0.05	1.00	0.001
	Africa	92	15.0	7.70–22.2	0	0.03	1.00	0.561
	Asia	642	0.6	0–1.2	0	0.01	1.00	0.059
	China *	90	0.5	−1.0–2.1	0	NA	NA	-
Cephalosporins	Cefamandole; cefazolin; cefepime; cefixime; cefoperazone; cefotaxime; cefoxitin; cefradine; ceftazidime; ceftiofur; ceftizoxime; ceftriaxone; cefuroxime; cephalexin; cephalothin; cephazolin	4481	23.8	16.2–31.3	0.083	99.7	336.89	0.210
	Africa	132	15.4	−1.5–32.2	0.020	90.43	10.45	<0.0001
	Asia	2552	25.0	14.7–35.2	0.089	99.71	340.82	<0.0001
	China	1659	16.2	6.1–26.3	0.047	99.55	221.02	<0.0001
	Europe/USA	138	63.5	12.7–114.3	0.199	99.18	122.35	<0.0001
Folate pathway inhibitors/sulfonamides	Sulfadiazine; sulfamethoxazole; sulfisoxazole; trimethoprim; trimethoprim-sulfamethoxazole	2356	26.1	13.9–38.3	0.075	99.55	224.17	<0.0001
	Africa *	92	36.7	26.9–46.6	0.0	0.0	1.0	0.386
	Asia	652	24.1	4.8–43.4	0.104	99.72	359.28	<0.0001
	China	1566	29.4	9.0–49.8	0.064	99.16	124.03	<0.0001
	Europe/USA *	46	8.7	0.6–16.8	0.0	-	-	-
Glycopeptides	Vancomycin; novobiocin	219	60.7	19.6–101	0.129	98.12	53.21	<0.0001
	Asia	157	60.9	−10.2–132	0.260	98.89	90.44	<0.0001
	China *	62	59.7	19.6–71.9	0.0	-	-	-
Lincosamides *	Clindamycin	62	NA					
Macrolides	Azithromycin; erythromycin; medemycin	857	22.1	5.0–39.3	0.082	99.47	189.31	<0.0001
	Africa *	46	15.2	4.8–25.6	0.0	-	-	-
	Asia	283	41.3	9.3–73.3	0.129	98.84	86.40	<0.0001
	China	528	4.9	0.5–9.4	0.002	89.61	9.63	<0.0001
Nitrofurans	Nitrofurantoin	260	58.8	17.8–99.8	0.127	98.03	50.76	<0.0001
	Asia *	124	87.9	79.5–96.3	0.0	-	-	-
	China *	90	19.4	12.4–26.3	0.0	-	-	-
	Europe/USA *	46	70.0	49.9–90.1	0.0	-	-	-
Oxazolidines	Furazolidone	62	NA					
Phenicols	Chloramphenicol; florfenicol	2062	14.5	3.9–25.1	0.063	99.76	415.43	<0.0001
	Africa	86	10.6	−9.4–30.7	0.019	90.52	10.55	0.001
	Asia	699	1.1	0.3–1.9	0.0	5.40	1.06	0.007
	China	1185	32.8	4.8–60.8	0.142	99.84	634.79	<0.0001
	Europe/USA	92	5.0	−4.4–14.4	0.042	75.77	4.13	0.004
Polypeptides	Bacitracin; colistin; polymyxin B	381	31.2	−5.7–68.2	0.213	99.91	1058.5	<0.0001
	Africa	92	1.7	−0.9–4.3	0.0	0.03	1.00	0.328
	Asia	137	90.9	74.3–107.5	0.013	93.52	15.44	<0.0001
	China	152	0.6	−0.6–1.9	0.0	0.35	1.00	0.858
Quinolones	Ciprofloxacin; enrofloxacin; levofloxacin; nalidixic acid; norfloxacin; ofloxacin; pefloxacin	3395	4.4	2.3–6.5	0.004	96.02	25.15	<0.0001
	Africa	80	1.2	−1.2–3.6	0.0	0.0	1.00	1.000
	Asia	1819	3.1	1.7–4.5	0.001	81.06	5.28	<0.0001
	China	1496	4.9	0–9.7	0.009	98.21	55.79	<0.0001
Rifampicins *	Rifampicin	561	NA					
Tetracyclines	Doxycycline; minocycline; oxytetracycline; tetracycline	2625	6.0	2.0–10.0	0.011	98.71	77.68	<0.0001
	Asia	1188	8.2	1.1–15.4	0.023	99.24	131.64	<0.0001
	China	1437	1.6	0.6–2.5	0.0	40.70	1.69	0.008

NA: data not available; *: the observed data based on a single study.

## Data Availability

Data used in the manuscript for tables and figures are presented in the accompanying Supplementary Materials.

## Supplementary Materials

The following supporting information can be downloaded at: https://www.mdpi.com/article/10.3390/antibiotics13040370/s1, Table S1. Completed PRISMA 2020 checklist. Table S2. Search algorithms and an example of key terms used for identification of relevant publications in *V. parahaemolyticus*. Table S3: AMR and its determinants of *V. parahaemolyticus* isolated from shrimp among relevant studies (*n* = 32). Figure S1. Funnel plots of prevalence of AMR in *V. parahaemolyticus* in shrimp to assess potential bias stratified by antimicrobial classes: (A) aminoglycosides; (B) beta-lactamases; (C) carbapenems; (D) cephalosporins; (E) folate pathway inhibitors/sulfonamides; (F) glycopeptides; (G) macrolides; (H) nitrofurans; (I) phenicols; (J) polypeptides; (K) quinolones; (L) tetracyclines. A red vertical line represents the overall prevalence from meta-analysis, and the diagonal lines provide 95% C.I. Figure S2. The meta-analysis of prevalence of resistance determinants from *V. parahaemolyticus* isolates in shrimp using random-effects model with a 95% C.I.: (A) a forest plot representing the results in meta-analysis with size squares proportional to the weight assigned to the study; (B) a funnel plot of occurrence of resistance determinants in *V. parahaemolyticus* to assess potential bias.
