# Supplementary material for: Meta-Analysis and Systematic Review of Phenotypic and Genotypic Antimicrobial Resistance and Virulence Factors in Vibrio parahaemolyticus Isolated from Shrimp
Source: Antibiotics (Basel). 2024 Apr 17;13(4):370. doi: 10.3390/antibiotics13040370 (PMC11047358; doi:10.3390/antibiotics13040370)
Supplement: Supplementary file 1 [file antibiotics-13-00370-s001.zip › antibiotics-2908102-supplementary.pdf]

antibiotics MDPI

Table S1: Completed PRISMA 2020 checklist[1]

PRISMA 2020 Checklist

Section and Topic	Item #	Checklist item	Location where item is reported
**TITLE**			
Title	1	Identify the report as a systematic review.	1
**ABSTRACT**			
Abstract	2	See the PRISMA 2020 for Abstracts checklist.	1
**INTRODUCTION**			
Rationale	3	Describe the rationale for the review in the context of existing knowledge.	1
Objectives	4	Provide an explicit statement of the objective(s) or question(s) the review addresses.	2
**METHODS**			
Eligibility criteria	5	Specify the inclusion and exclusion criteria for the review and how studies were grouped for the syntheses.	13-14
Information sources	6	Specify all databases, registers, websites, organisations, reference lists and other sources searched or consulted to identify studies. Specify the date when each source was last searched or consulted.	14
Search strategy	7	Present the full search strategies for all databases, registers and websites, including any filters and limits used.	14, Table S3
Selection process	8	Specify the methods used to decide whether a study met the inclusion criteria of the review, including how many reviewers screened each record and each report retrieved, whether they worked independently, and if applicable, details of automation tools used in the process.	14
Data collection process	9	Specify the methods used to collect data from reports, including how many reviewers collected data from each report, whether they worked independently, any processes for obtaining or confirming data from study investigators, and if applicable, details of automation tools used in the process.	14
Data items	10a	List and define all outcomes for which data were sought. Specify whether all results that were compatible with each outcome domain in each study were sought (e.g. for all measures, time points, analyses), and if not, the methods used to decide which results to collect.	14-15
	10b	List and define all other variables for which data were sought (e.g. participant and intervention characteristics, funding sources). Describe any assumptions made about any missing or unclear information.	14-15
Study risk of bias assessment	11	Specify the methods used to assess risk of bias in the included studies, including details of the tool(s) used, how many reviewers assessed each study and whether they worked independently, and if applicable, details of automation tools used in the process.	15
Effect measures	12	Specify for each outcome the effect measure(s) (e.g. risk ratio, mean difference) used in the synthesis or presentation of results.	NA
Synthesis methods	13a	Describe the processes used to decide which studies were eligible for each synthesis (e.g. tabulating the study intervention characteristics and comparing against the planned groups for each synthesis (item #5)).	13-14
	13b	Describe any methods required to prepare the data for presentation or	14

Section and Topic	Item #	Checklist item	Location where item is reported
		synthesis, such as handling of missing summary statistics, or data conversions.	
	13c	Describe any methods used to tabulate or visually display results of individual studies and syntheses.	15
	13d	Describe any methods used to synthesize results and provide a rationale for the choice(s). If meta-analysis was performed, describe the model(s), method(s) to identify the presence and extent of statistical heterogeneity, and software package(s) used.	15
	13e	Describe any methods used to explore possible causes of heterogeneity among study results (e.g. subgroup analysis, meta-regression).	15
	13f	Describe any sensitivity analyses conducted to assess robustness of the synthesized results.	15
Reporting bias assessment	14	Describe any methods used to assess risk of bias due to missing results in a synthesis (arising from reporting biases).	15
Certainty assessment	15	Describe any methods used to assess certainty (or confidence) in the body of evidence for an outcome.	15
**RESULTS**			
Study selection	16a	Describe the results of the search and selection process, from the number of records identified in the search to the number of studies included in the review, ideally using a flow diagram.	2-3, Figure 1
	16b	Cite studies that might appear to meet the inclusion criteria, but which were excluded, and explain why they were excluded.	2-3
Study characteristics	17	Cite each included study and present its characteristics.	3-4
Risk of bias in studies	18	Present assessments of risk of bias for each included study.	Figure 2C, 2D, S1 & S2
Results of individual studies	19	For all outcomes, present, for each study: (a) summary statistics for each group (where appropriate) and (b) an effect estimate and its precision (e.g. confidence/credible interval), ideally using structured tables or plots.	3-6 & Table 2
Results of syntheses	20a	For each synthesis, briefly summarise the characteristics and risk of bias among contributing studies.	9, Figure 2C, 2D, S1 & S2
	20b	Present results of all statistical syntheses conducted. If meta-analysis was done, present for each the summary estimate and its precision (e.g. confidence/credible interval) and measures of statistical heterogeneity. If comparing groups, describe the direction of the effect.	4-8 & Figure 2
	20c	Present results of all investigations of possible causes of heterogeneity among study results.	Figure 2
	20d	Present results of all sensitivity analyses conducted to assess the robustness of the synthesized results.	Table 1
Reporting biases	21	Present assessments of risk of bias due to missing results (arising from reporting biases) for each synthesis assessed.	Figure S1
Certainty of evidence	22	Present assessments of certainty (or confidence) in the body of evidence for each outcome assessed.	Table 2 & Figure 2
**DISCUSSION**			

Section and Topic	Item #	Checklist item	Location where item is reported
Discussion	23a	Provide a general interpretation of the results in the context of other evidence.	11-12
	23b	Discuss any limitations of the evidence included in the review.	13
	23c	Discuss any limitations of the review processes used.	13
	23d	Discuss implications of the results for practice, policy, and future research.	13
**OTHER INFORMATION**			
Registration and protocol	24a	Provide registration information for the review, including register name and registration number, or state that the review was not registered.	13
	24b	Indicate where the review protocol can be accessed, or state that a protocol was not prepared.	13
	24c	Describe and explain any amendments to information provided at registration or in the protocol.	13-14
Support	25	Describe sources of financial or non-financial support for the review, and the role of the funders or sponsors in the review.	16
Competing interests	26	Declare any competing interests of review authors.	16
Availability of data, code and other materials	27	Report which of the following are publicly available and where they can be found: template data collection forms; data extracted from included studies; data used for all analyses; analytic code; any other materials used in the review.	16

[1] The PRISMA checklist was retrieved from Page et al.,2021.
For more information, visit: http://www.prisma-statement.org/

Table S2: Search algorithms and example of key terms used for identification of relevant publications in *V. parahaemolyticus*

Search algorithm	Example
Bacteria	*Vibrio parahaemolyticus*; *V. parahaemolyticus*
Target animal	Shrimp
Antimicrobial resistance	Antimicrobial resistance; AMR; antibiotic resistance; antimicrobial susceptibility test; resistance profile
Virulence factor	Virulence, TDH, TRH, pathogenicity
Bacteria * target animal	*Vibrio parahaemolyticus* (*V. parahaemolyticus*) * shrimp
Bacteria * virulence factor	*V. parahaemolyticus* * virulence; *V. parahaemolyticus* * TDH; *V. parahaemolyticus* * TRH; *V. parahaemolyticus* * pathogenicity
Bacteria * target animal * antimicrobial resistance	*V. parahaemolyticus* * shrimp * antimicrobial resistance; *V. parahaemolyticus* * shrimp * AMR; *V. parahaemolyticus* * shrimp * antibiotic resistance; *V. parahaemolyticus* * shrimp * antimicrobial susceptibility test; *V. parahaemolyticus* * shrimp * antimicrobial susceptibility testing; *V. parahaemolyticus* * shrimp * resistance profile

Table S3: AMR and their determinants of *V. parahaemolyticus* isolated from shrimp among relevant studies (n = 32)

ID	Author and published year	Country	AST	Antimicrobial class	Antimicrobial	Prevalence (%)
1	Mulya et al. (2022)	Indonesia	Disc diffusion (CLSI 2016)	Beta-lactams	Ampicillin	100.0
				Phenicols	Chloramphenicol	0
				Quinolones	Ciprofloxacin	0
					Enrofloxacin	0
				Tetracyclines	Tetracycline	41.7
2	Dang et al. (2022)	Vietnam	Disc diffusion (CLSI M100-S24, CLSI, 2014) and broth macro dilution (NCCLS)	Aminoglycosides	Gentamicin	46.6
					Kanamycin	34.5
					Neomycin	39.7
				Beta-lactams	Amoxicillin	100.0
				Cephalosporins	Cephalexin	100.0
				Folate pathway inhibitor/sulfonamides	Sulfadiazine	94.8
				Macrolides	Erythromycin	88.0
				Tetracyclines	Doxycycline	3.5
					Oxytetracycline	36.2
3	Nadella et al (2022)	India	Disc diffusion (CLSI, 2020)	Folate pathway inhibitor/sulfonamides	Tri-sulfa	75.0
				Macrolides	Erythromycin	70.0
				Phenicol	Chloramphenicol	40.0
				Quinolones	Ciprofloxacin	70.0
				Tetracyclines	Oxytetracycline	95.0
4	Babu et al. (2021)	India	Disc diffusion	NA	NA	NA
5	Vu et al. (2022)	Vietnam	Disc diffusion (CLSI, 2015)	Aminoglycosides	Gentamicin	0
				Beta-lactams	Ampicillin	80.8
				Carbapenems	Imipenem	0
				Cephalosporins	Cefotaxime	11.5
					Cefoxitin	0
					Ceftazidime	11.5
				Folate pathway inhibitor/sulfonamides	Tri-sulfa	7.7
				Phenicol	Chloramphenicol	0
				Quinolones	Ciprofloxacin	0
				Tetracyclines	Tetracycline	3.9

6	Haifa-Haryani et al. (2022)	Malaysia	Disc Diffusion (CLSI, M45-A2)	Aminoglycosides	Gentamicin	7.3
					Kanamycin	40.3
				Beta-lactams	Ampicillin	84.7
					Penicillin	96.8
				Cephalosporins	Cefepime	1.6
					Cefotaxime	73.4
					Ceftazidime	24.2
					Cephalothin	86.3
				Folate pathway inhibitor/sulfonamides	Sulfamethoxazole	13.7
				Glycopeptides	Vancomycin	96.8
				Macrolides	Erythromycin	19.4
				Nitrofurans	Nitrofurantoin	53.2
				Phenicol	Chloramphenicol	8.9
				Quinolones	Ciprofloxacin	5.7
					Norfloxacin	0.8
				Tetracyclines	Tetracycline	0.8
7	Kim et al. (2021)	Korea	Broth micro dilution Sensititre® (CLSI, 2016; EUCAST, 2019)	Aminoglycosides	Amikacin	4.2
					Gentamicin	0
					Streptomycin	91.7
				Beta-lactams	Ampicillin	100.0
				Carbapenems	Imipenem	0
				Cephalosporins	Cefotaxime	0
					Cefoxitin	0
					Ceftazidime	4.2
					Ceftriaxone	2.1
				Folate pathway inhibitor/sulfonamides	Tri-sulfa	6.3
				Macrolides	Azithromycin	0
				Phenicol	Chloramphenicol	0
				Polymyxins	Colistin	100.0
				Quinolones	Ciprofloxacin	0
					Nalidixic acid	0
				Tetracyclines	Tetracycline	6.3
8	Mok et al. (2021)	Korea	Broth micro dilution Sensititre®	Aminoglycosides	Gentamicin	0
					Streptomycin	3.4
				Beta-lactams	Amox/cla	2.3
					Ampicillin	11.2
				Carbapenems	Meropenem	0
				Cephalosporins	Cefepime	0
					Cefoxitin	0
					Ceftazidime	0
					Ceftiofur	0
				Folate pathway inhibitor/sulfonamides	Tri-sulfa	0
				Phenicols	Chloramphenicol	0
				Polymyxins	Colistin	82.0
				Quinolones	Ciprofloxacin	0
					Nalidixic acid	0
				Tetracyclines	Tetracycline	0

9	Jin et al. (2021)	China	Broth micro-dilution (CLSI, 2019)	Aminoglycosides	Kanamycin	1.4
				Beta-lactams	Amox/cla	91.9
					Ampicillin	94.6
				Cephalosporins	Cefazolin	1.4
					Cefuroxime	6.8
				Phenicol	Chloramphenicol	98.7
				Quinolones	Ciprofloxacin	2.7
					Nalidixic acid	4.1
					Norfloxacin	0
10	Siddique et al. (2021)	Bangladesh	Broth micro-dilution (CLSI, 2019)	Aminoglycosides	Gentamicin	11.8
					Streptomycin	5.9
				Beta-lactams	Amoxicillin	94.1
					Ampicillin	94.1
					Piper/tazo	0
				Carbapenems	Imipenem	0
					Meropenem	0
				Cephalosporins	Cefixime	0
					Cefotaxime	29.4
					Cefoxitin	0
					Ceftriaxone	17.7
				Folate pathway inhibitor/sulfonamides	Tri-sulfa	0
				Quinolones	Ciprofloxacin	0
					Nalidixic acid	0
				Tetracyclines	Tetracycline	0
11	Álvarez-Contreras et al. (2021)	Mexico	Micro dilution (CLSI, 2012; EP17-A2)	Aminoglycosides	Amikacin	41.3
					Gentamicin	21.7
					Netilmicin	52.2
				Beta-lactams	Ampicillin	100.0
					Carbenicillin	28.3
				Cephalosporins	Cefotaxime	78.3
					Cephalothin	100.0
					Ceftriaxone	13.0
				Folate pathway inhibitor/sulfonamides	Tri-sulfa	8.7
				Nitrofurans	Nitrofurantoin	21.7
				Phenicols	Chloramphenicol	0
				Quinolones	Pefloxacin	10.9
12	Janecko et al (2021)	UK	NA	NA	NA	NA
13	Venggadasamy et al. (2021)	Malaysia	Disk diffusion (CLSI, 2016)	Aminoglycosides	Amikacin	2.3
					Gentamicin	4.7
					Kanamycin	9.3
				Beta-lactams	Ampicillin	95.4
					Ampi/sul	81.4
				Carbapenems	Imipenem	23.3
				Cephalosporins	Cefotaxime	37.2
					Ceftazidime	7.0
				Folate pathway inhibitor/sulfonamides	Tri-sulfa	0
				Phenicols	Chloramphenicol	0

				Quinolones	Levofloxacin	2.3
					Nalidixic acid	2.3
				Tetracyclines	Oxytetracycline	16.3
					Tetracycline	18.6
14	Yasin et al. (2021)	Bangladesh	Disk diffusion (CLSI, 2013)	Aminoglycosides	Gentamicin	100.0
				Beta-lactams	Ampicillin	0
					Penicillin	100.0
				Cephalosporins	Ceftazidime	0
					Ceftriaxone	0
				Folate pathway inhibitor/sulfonamides	Trimethoprim	0
					Tri-sulfa	100.0
				Macrolides	Azithromycin	0
					Erythromycin	0
				Phenicols	Chloramphenicol	0
				Quinolones	Ciprofloxacin	0
				Tetracyclines	Tetracycline	100.0
15	Nguyen et al. (2020)	Vietnam	Disk-diffusion method (CLSI, 2018)	Aminoglycosides	Amikacin	1.6
					Gentamicin	0
				Beta-lactams	Amox/cla	9.4
				Carbapenems	Ampicillin	100.0
				Cephalosporins	Imipenem	3.1
					Cefotaxime	31.3
				Folate pathway inhibitor/sulfonamides	Ceftazidime	15.6
					Tri-sulfa	26.6
				Phenicols	Chloramphenicol	1.6
				Quinolones	Ciprofloxacin	11.0
					Ofloxacin	9.4
				Tetracyclines	Tetracycline	10.9
16	Navaneeth et al. (2020)	India	Disk-diffusion method	Aminoglycosides	Amikacin	0
					Gentamicin	0
				Beta-lactams	Amox/clav	40.0
					Amp/sul	2.9
					Ticarcillin	4.8
				Carbapenems	Meropenem	0
				Cephalosporins	Cefotaxime	9.5
					Cephalothin	0
				Phenicols	Chloramphenicol	1.9
				Quinolones	Ciprofloxacin	1.9
					Levofloxacin	1.9
					Nalidixic acid	0
					Norfloxacin	0
					Ofloxacin	2.9
				Tetracyclines	Minocycline	1.9
					Tetracycline	100.0
17	Jiang et al. (2020)	China	Disk-diffusion method (CLSI, 2017)	Aminoglycosides	Streptomycin	74.4
				Beta-lactams	Ampicillin	76.7
				Carbapenems	Imipenem	0
				Cephalosporins	Cefamandole	18.9
					Cefazolin	25.6
					Cefepime	3.33
					Ceftizoxime	4.44

				Folate pathway inhibitor/sulfonamides	Sulfisoxazole	47.8
					Tri-sulfa	64.4
				Nitrofurans	Nitrofurantoin	0
				Phenicol	Chloramphenicol	57.8
					Florfenicol	53.3
				Polymyxins	Polymyxin B	0
				Quinolones	Ciprofloxacin	0
					Enrofloxacin	47.8
					Norfloxacin	0
				Tetracyclines	Doxycycline	46.7
					Tetracycline	57.8
18	Beshiru et al. (2020)	Nigeria	Disk-diffusion method	Aminoglycosides	Gentamicin	0
					Kanamycin	4.4
					Streptomycin	23.9
					Tobramycin	0
				Beta-lactams	Amoxicillin	82.6
					Amox-clav	23.9
					Ampicillin	6.5
					Amp/sul	23.9
					Penicillin	87.0
				Carbapenems	Imipenem	13.0
					Meropenem	17.4
				Cephalosporins	Cefotaxime	30.4
					Cephalothin	17.4
				Folate-pathway inhibitor/sulfonamides	Sulfamethoxazole	32.6
					Trimethoprim	41.3
				Macrolides	Erythromycin	15.2
				Phenicols	Chloramphenicol	21.7
				Polymyxins	Colistin	0
					Polymyxin B	4.4
					Ciprofloxacin	15.2
				Quinolones	Ofloxacin	19.6
					Doxycycline	41.3
				Tetracyclines	Oxytetracycline	37.0
					Tetracycline	28.3
19	Rahman et al. (2020)	Bangladesh	Disk-diffusion method	Aminoglycosides	Gentamicin	0
					Kanamycin	0
				Beta-lactams	Amoxicillin	75.0
					Ampicillin	56.3
				Folate-pathway inhibitor/sulfonamides	Sulfamethoxazole	0
				Macrolides	Azithromycin	0
					Erythromycin	6.3
				Nitrofurans	Nitrofurantoin	0
				Phenicols	Chloramphenicol	0
				Polymyxins	Polymyxin B	31.3
				Quinolones	Ciprofloxacin	0
				Tetracyclines	Tetracycline	0
20	Li et al. (2020)	China	Disk-diffusion method (CLSI, 2018)	Aminoglycosides	Gentamicin	20.8
					Kanamycin	44.6
					Streptomycin	65.8

				Beta-lactams	Ampicillin	79.2
				Cephalosporins	Cefazolin	58.9
					Cephalothin	74.8
				Folate-pathway inhibitor/sulfonamides	Tri-sulfa	10.4
				Macrolides	Azithromycin	12.9
				Phenicols	Chloramphenicol	4.0
				Quinolones	Ciprofloxacin	3.5
					Nalidixic acid	2.5
				Tetracyclines	Tetracycline	14.4
21	Zangoei-Fard et al. (2020)	Iran	Disk-diffusion method (CLSI, 2010)	Aminoglycosides	Gentamicin	68.4
					Streptomycin	42.1
					Vancomycin	36.8
				Beta-lactams	Ampicillin	57.9
					Penicillin	47.4
				Cephalosporins	Cefotaxime	31.6
					Cephalothin	36.8
				Folate-pathway inhibitor/sulfonamides	Tri-sulfa	36.8
				Macrolides	Azithromycin	26.3
					Erythromycin	47.4
				Quinolones	Ciprofloxacin	21.1
					Nalidixic acid	21.1
				Tetracyclines	Tetracycline	52.6
22	Hu et al. (2020)	China	Disk-diffusion method (CLSI, 2015)	Aminoglycosides	Amikacin	0
					Gentamicin	4.8
					Kanamycin	0
					Neomycin	0
				Beta-lactams	Ampicillin	66.1
					Carboxybenzicillin	0
					Oxacillin	71.0
					Penicillin	77.4
					Piperacillin	0
				Cephalosporins	Cefazolin	0
					Cefoperazone	0
					Cefradine	17.7
					Ceftazidime	0
					Ceftriaxone	0
					Cefuroxime	19.4
					Cephalexin	17.7
				Folate-pathway inhibitor/sulfonamides	Tri-sulfa	6.5
				Glycopeptides	Vancomycin	59.7
				Lincosamide	Clindamycin	8.1
				Macrolides	Erythromycin	0
					Medemycin	0
				Oxazolidines	Furazolidone	0
				Phenicols	Chloramphenicol	11.3
				Polymyxins	Polymyxin	0
				Quinolones	Ciprofloxacin	0
					Norfloxacin	0

					Ofloxacin	0
				Tetracyclines	Doxycycline	8.1
					Minocycline	0
					Tetracycline	6.5
23	Lu et al. (2020)	NA	NA	NA	NA	NA
24	Bughe et al. (2020)	Cameroon	Disk-diffusion method (CLSI, 2016)	Aminoglycosides	Netilmicin	0
					Streptomycin	0
				Beta-lactams	Penicillin	80.0
				Cephalosporins	Ceftazidime	0
				Phenicols	Chloramphenicol	0
				Quinolones	Ciprofloxacin	0
					Norfloxacin	0
25	Tan et al. (2020)	Malaysia	Disk-diffusion method (CLSI, 2010)	Aminoglycosides	Amikacin	37.5
					Gentamicin	6.7
				Beta-lactams	Amox/cla	0.8
					Ampicillin	84.2
					Amp/sul	0.8
					Penicillin	100.0
					Piperacillin	35.8
					Piper/tazo	15.8
				Carbapenems	Imipenem	0
					Meropenem	0
				Cephalosporins	Cefazolin	84.2
					Cefepime	3.3
					Cefotaxime	5.0
					Cefoxitin	12.5
					Ceftazidime	5.0
					Cefuroxime	51.7
					Cephalothin	54.2
				Folate-pathway inhibitor/sulfonamides	Tri-sulfa	0
				Phenicols	Chloramphenicol	0
				Quinolones	Ciprofloxacin	13.3
					Levofloxacin	1.7
					Ofloxacin	2.5
				Tetracyclines	Doxycycline	0
					Tetracycline	0
26	Hong et al. (2020)	Vietnam	Disk-diffusion method (CLSI, 2014)	Aminoglycosides	Gentamicin	0
					Kanamycin	0
					Streptomycin	91.7
				Beta-lactams	Ampicillin	91.7
				Carbapenems	Tebipenem	0
				Phenicols	Chloramphenicol	0
				Polymyxins	Colistin	91.7
				Quinolones	Nalidixic acid	8.3
					Ofloxacin	0
				Tetracyclines	Oxytetracycline	25.0
27	Tengfei et al. (2020)	China	Disc diffusion method (CLSI, 2018)	Aminoglycosides	Gentamicin	4.0
					Kanamycin	50.5
					Streptomycin	91.1
				Beta-lactams	Ampicillin	58.4

					Piperacillin	36.6
				Cephalosporins	Cephazolin	42.6
				Macrolides	Azithromycin	7.9
					Erythromycin	4.0
				Quinolones	Ciprofloxacin	5.0
					Nalidixic acid	4.0
				Tetracyclines	Minocycline	3.0
					Tetracycline	4.0
28	Lei et al. (2020)	China	Broth microdilution method	Aminoglycosides	Gentamicin	18.9
				Beta-lactams	Ampicillin	83.0
				Cephalosporins	Cefotaxime	0
					Ceftazidime	0
				Phenicols	Chloramphenicol	4.7
				Quinolones	Ciprofloxacin	4.7
					Levofloxacin	4.7
				Tetracyclines	Tetracycline	15.1
29	Amin et al. (2020)	Bangladesh	Disc diffusion method	Aminoglycosides	Streptomycin	0
				Folate-pathway inhibitor/sulfonamides	Tri-sulfa	42.4
				Glycopeptides	Novobiocin	24.2
				Macrolides	Azithromycin	30.3
				Phenicols	Chloramphenicol	0
				Quinolones	Ciprofloxacin	0
30	Narayanan et al. (2020)	India	Disc diffusion method	Aminoglycosides	Gentamicin	3.7
				Beta-lactams	Amox/cla	7.4
					Ampicillin	100.0
				Carbapenems	Meropenem	3.7
				Cephalosporins	Cefepime	44.4
					Cefotaxime	74.1
					Cefoxitin	48.1
					Ceftazidime	29.6
				Folate-pathway inhibitor/sulfonamides	Tri-sulfa	0
				Phenicols	Chloramphenicol	0
				Quinolones	Ciprofloxacin	3.7
				Tetracyclines	Tetracycline	0
31	Amatul-Samahah et al. (2020)	Malaysia	Disk-diffusion method (CLSI, 2015)	Aminoglycosides	Gentamicin	0
					Kanamycin	0
					Streptomycin	0
				Beta-lactams	Ampicillin	50.0
				Cephalosporins	Cefepime	0
					Ceftriaxone	0
					Cephalothin	0
				Glycopeptides	Vancomycin	100.0
				Macrolides	Erythromycin	0
				Phenicols	Chloramphenicol	0
				Polymyxins	Bacitracin	100.0
				Quinolones	Ciprofloxacin	0
				Tetracyclines	Tetracycline	0

32	Su et al. (2020)	China	Disk-diffusion method (CLSI, 2012)	Aminocyclitol	Spectinomycin	44.6
				Aminoglycosides	Gentamicin	37.1
					Kanamycin	50.1
					Streptomycin	75.4
				Beta-lactams	Ampicillin	93.0
				Folate-pathway inhibitor/sulfonamides	Trimethoprim	44.0
					Tri-sulfa	5.0
				Phenicols	Chloramphenicol	0.5
				Rifamycins	Rifampicin	82.9
				Tetracyclines	Tetracycline	24.1

Amox/cla: Amoxicillin/clavulanic acid; Ampi/sul: Ampicillin-sulbactam; Piper/tazo: Piperacillin-tazobactam; Tri-sulfa: Trimethoprim-sulfamethoxazole; NA: not available data

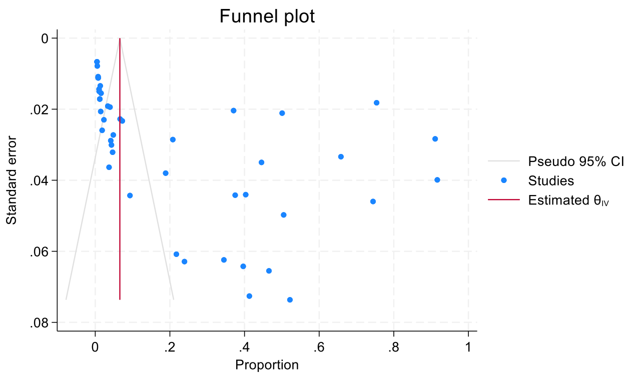

(A) Aminoglycosides

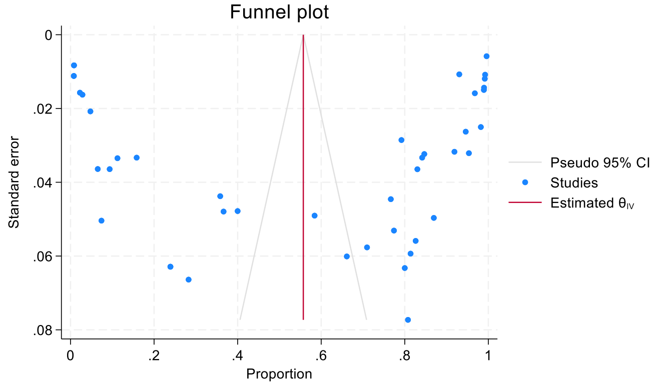

(B) Beta-lactams

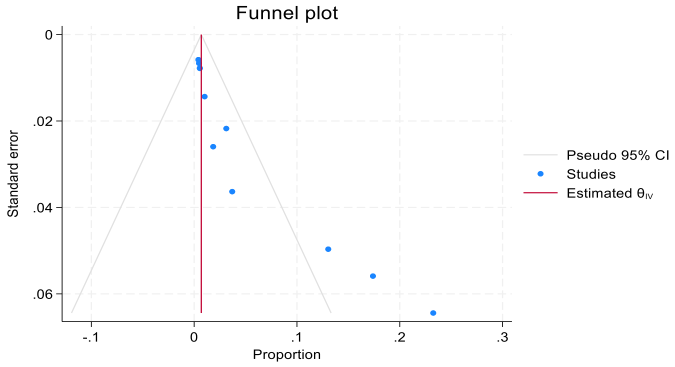

(C) Carbapenems

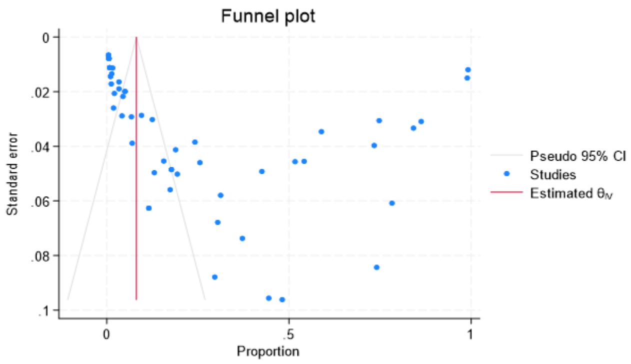

(D) Cephalosporins

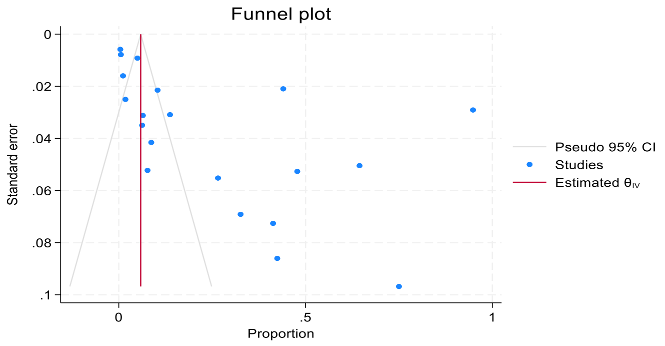

(E) Folate pathway inhibitors/sulfonamides

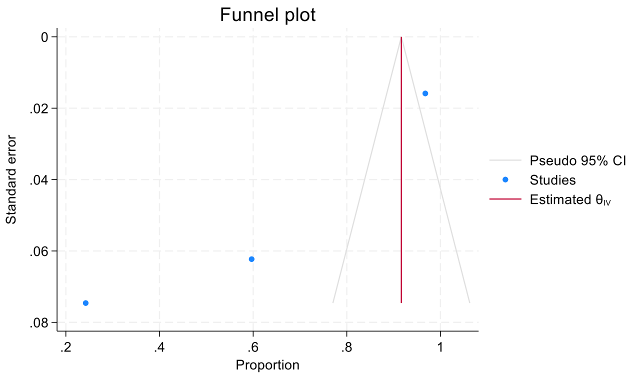

(F) Glycopeptides

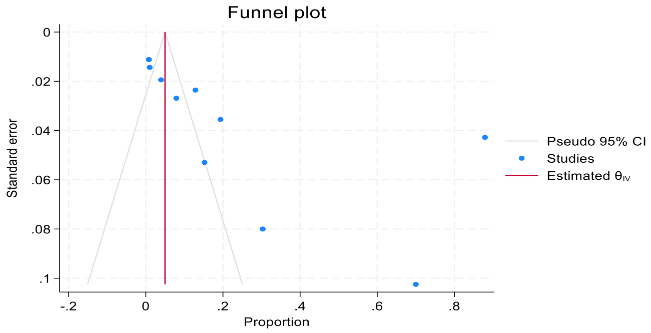

(G) Macrolides

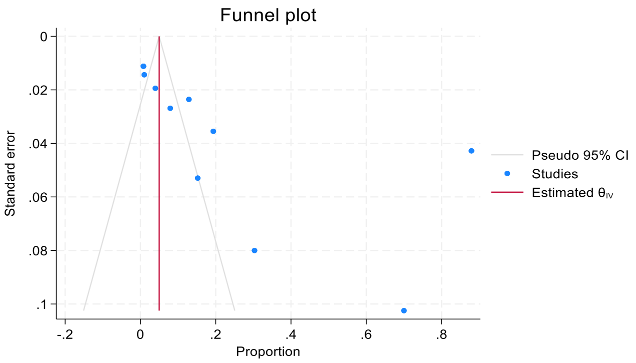

(H) Nitrofurans

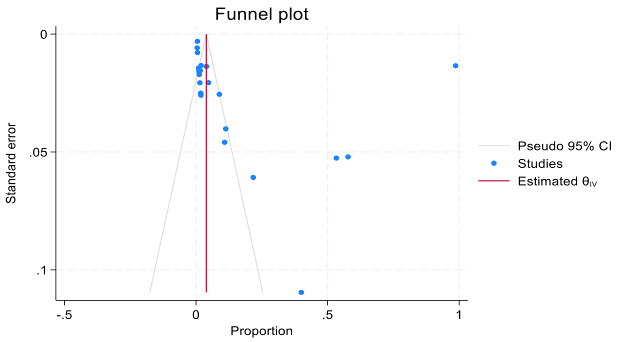

(I) Phenicols

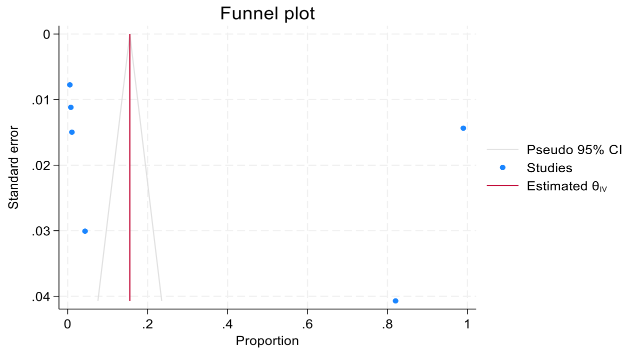

(J) Polypeptides

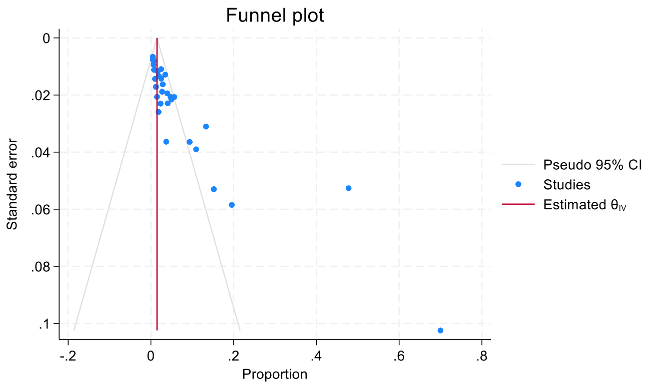

(K) Quinolones

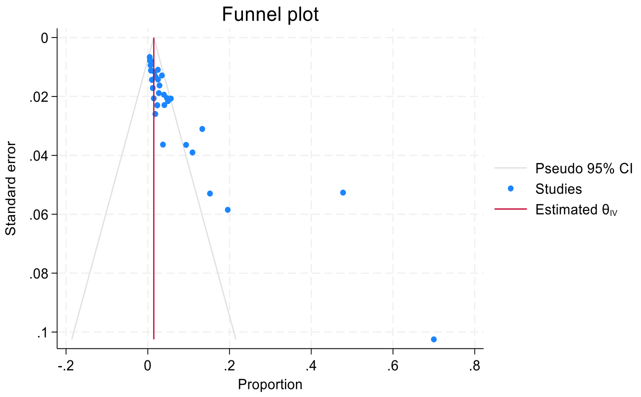

(L) Tetracyclines

Figure S1. Funnel plots of prevalence of AMR in *V. parahaemolyticus* in shrimp to assess potential bias stratified by antimicrobial classes: (A) aminoglycosides; (B) beta-lactamases; (C) carbapenems; (D) cephalosporins; (E) folate pathway inhibitors/sulfonamides; (F) glycopeptides; (G) macrolides; (H) nitrofurans; (I) phenicols; (J) polypeptides; (K) Quinolones; (L) Tetracyclines, and a red vertical line represents the overall prevalence from meta-analysis and the diagonal lines provide 95% C.I.

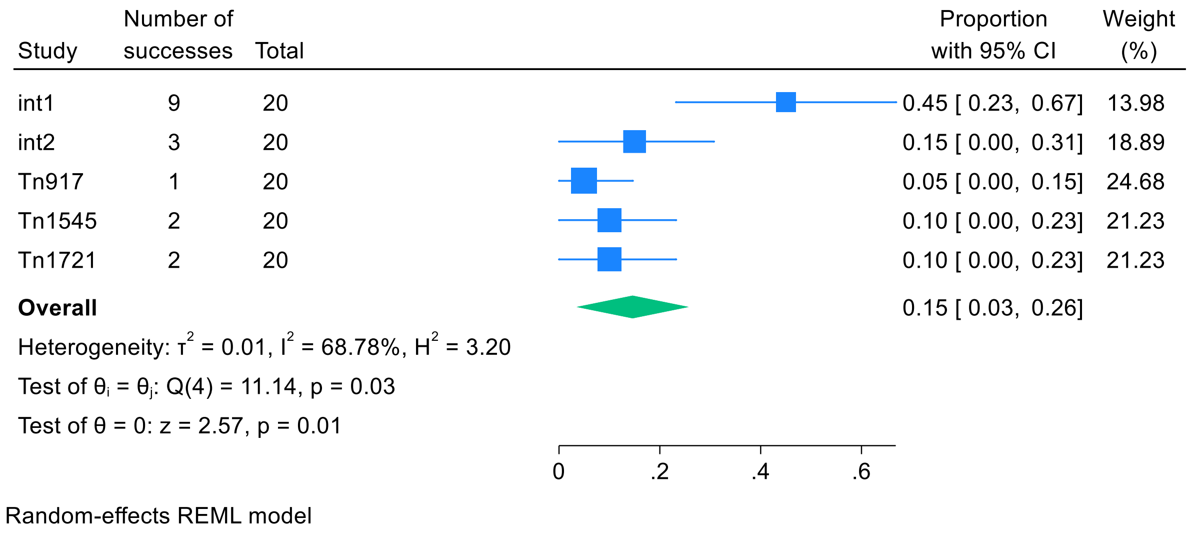

(A)

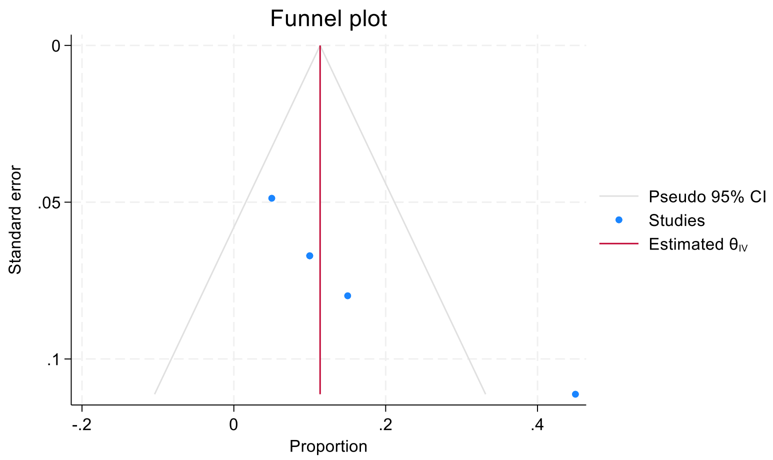

(B)

Figure S2. The meta-analysis of prevalence of resistance determinants from *V. parahaemolyticus* isolates in shrimp using random-effects model with a 95% C.I.; (A) a forest plot representing the results in meta-analysis with size squares proportional to the weight assigned to the study; (B) a funnel plot of occurrence of resistance determinants in *V. parahaemolyticus* to assess potential bias.
